# Shape Memory Alloy (SMA) Actuator With Embedded Liquid Metal Curvature Sensor for Closed-Loop Control

**DOI:** 10.3389/frobt.2021.599650

**Published:** 2021-03-11

**Authors:** Zhijian Ren, Masoud Zarepoor, Xiaonan Huang, Andrew P. Sabelhaus, Carmel Majidi

**Affiliations:** ^1^ Department of Mechanical Engineering, Carnegie Mellon University, Pittsburgh, PA, United States; ^2^ School of Engineering and Technology, Lake Superior State University, Sault Ste Marie, MI, United States; ^3^ Robotics Institute, Carnegie Mellon University, Pittsburgh, PA, United States

**Keywords:** soft robotics, liquid metal, shape memory alloy, soft sensors, soft actuators, closed-loop control

## Abstract

We introduce a soft robot actuator composed of a pre-stressed elastomer film embedded with shape memory alloy (SMA) and a liquid metal (LM) curvature sensor. SMA-based actuators are commonly used as electrically-powered limbs to enable walking, crawling, and swimming of soft robots. However, they are susceptible to overheating and long-term degradation if they are electrically stimulated before they have time to mechanically recover from their previous activation cycle. Here, we address this by embedding the soft actuator with a capacitive LM sensor capable of measuring bending curvature. The soft sensor is thin and elastic and can track curvature changes without significantly altering the natural mechanical properties of the soft actuator. We show that the sensor can be incorporated into a closed-loop “bang-bang” controller to ensure that the actuator fully relaxes to its natural curvature before the next activation cycle. In this way, the activation frequency of the actuator can be dynamically adapted for continuous, cyclic actuation. Moreover, in the special case of slower, low power actuation, we can use the embedded curvature sensor as feedback for achieving partial actuation and limiting the amount of curvature change.

## Introduction

Soft robots have unique potential in bio-inspired robotics due to their ability to mimic the natural mechanics, deformability, and mobility of soft biological organisms (Li et al., 2011; Ijspeert, 2014; Aguilar et al., 2016; Zimmerman and Abdelkefi, 2017; Majidi, 2018; Rich et al., 2018). Legged soft robots have been built and utilized for walking on different terrestrial surfaces and maneuvering through very tight spaces and openings (Shepherd et al., 2011; Drotman et al., 2017). The performance of soft legged robots highly depends on the soft limb actuators (Tolley et al., 2014; Duduta et al., 2017; Huang et al., 2019a) used in the soft robot. To achieve robust legged-locomotion for a soft robot, the limb actuators should have two seemingly paradoxical features: sufficiently high bending stiffness in order to support the robot’s weight and push the robot forward during walking and adequate mechanical compliance to enable deformation through confined spaces. As in natural organisms, this combination of load-bearing stiffness and compliance is achieved with rigidity-tuning actuators that transition between a compliant, malleable state and a stiff, shape-restoring state.

There are a variety of actuator designs that allow for combined shape and stiffness tuning, ranging from pneumatic artificial muscles to hydraulically-amplified electrostatic actuators (Hines et al., 2017; Boyraz et al., 2018; Coyle et al., 2018; Gu et al., 2018; Kellaris et al., 2018). Among these, shape memory alloys (SMAs), such as nickel-titanium (nitinol), are especially popular due to their high work density, versatile form factor, and ability to be electrically activated using miniaturized, on-board power and electronics (Mohd Jani et al., 2014; Elahinia, 2015; Rodrigue et al., 2017). However, SMA actuators can be difficult to operate, especially at frequencies greater than 0.1 Hz. This is because SMA must be heated in order to induce the shape memory phase transition and allowed to cool back to room temperature for the actuator to return to its natural shape. If reactivated too quickly, the actuator has the potential for permanent changes in the nitinol crystal structure due to overheating. Closed-loop strategies for operating SMA actuators based on measuring changes in the electrical resistance (Ikuta et al., 1988; Maffiodo and Raparelli, 2016) and temperature (Kuribayashi, 1991; Jin et al., 2016b) of the nitinol wire have been proposed. However, soft robot locomotion with SMA sometimes requires fast push-off motions and very short activation time to achieve a relatively high speed. This makes it difficult to track the change in temperature or electrical resistance of SMA wire during actuation. Besides, it is extremely difficult to accurately measure the temperature of SMA wire due to its small diameter (<1 mm) and the unpredictable heat transfer with surrounding material and environment. Thus, a new closed-loop strategy based on a different type of measurement is needed for the continuous, cyclic actuation of SMA-based soft robots.

**FIGURE 1:**
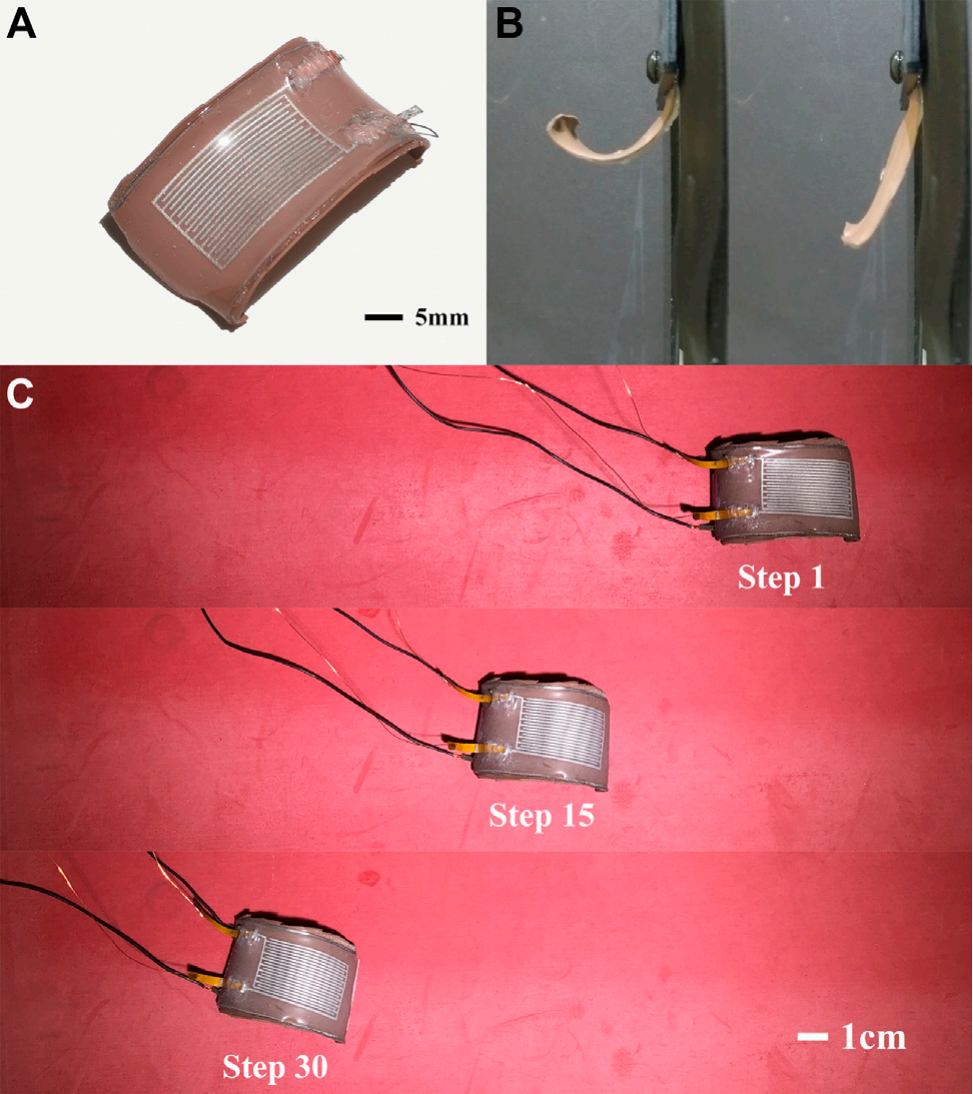
**(A)** Sensorized SMA actuator composed of thermally conductive elastomers, U-shaped SMA wire and LM strain gauge. **(B)** The relaxed **(left)** and activated **(right)** state of the actuator. **(C)** Locomotion of single actuator with closed-loop control using sensor feedback.

Here, we address this challenge by introducing a sensorized SMA actuator (Figure 1A) in which a thermally conductive elastomer is embedded with a nitinol wire and a soft capacitive strain gauge. The strain gauge is composed of microfluidic channels of liquid metal (LM) alloy embedded in a soft silicone elastomer. Because it is fluidic, the embedded LM strain gauge has negligible influence on the natural stiffness and mechanics of the actuator. This is in contrast to other soft sensor architectures that utilize percolating networks of conductive filler particles embedded within a stretchable elastomer matrix (Wagner and Bauer, 2012). There have been numerous studies on the use of LM alloys for soft sensors (Dickey, 2017). Other studies have also looked at the use of ionic fluids and ionic gels (Chossat et al., 2013; Lee et al., 2018; Gu et al., 2019).

The actuator consists of a U-shape nitinol wire that is embedded between a pair of unstretched and stretched elastomer layers. It is able to change reversibly between an unactuated soft curled shape and an actuated rigid straightened shape (Figure 1B). Previously, it was shown that flexural SMA actuators could enable an untethered soft quadruped (SEAQ) to walk with a maximum speed of 0.56 body lengths per second (Huang et al., 2019b). However, SEAQ did not contain any sensors to track actuator relaxation and the limbs were instead cyclically activated using a fixed cooling time. The absence of sensing indicates that during locomotion, the robot is incapable of monitoring whether it is activated either prematurely (i.e., before the limb can recover to its natural curvature) or too infrequently (with excessive pause between activation cycles). Here, we overcome this by using the embedded LM strain gauge to track the limb curvature and applying a control scheme to ensure the full recovery of the limb to its natural curled compliant shape before the next actuation cycle (Figure 1C).

We use eutectic gallium-indium (EGaIn) as the liquid metal alloy due to its high electrical conductivity, low viscosity, and compatibility with airbrush-based stencil lithography (Jeong et al., 2012, Jeong et al., 2015; Wissman et al., 2013). Elastomer films embedded with microfluidic channels of EGaIn have previously been used to create flexural sensors that change electrical resistance or capacitance in response to bending (Majidi et al., 2011; Matsuzaki and Tabayashi, 2015; Lu et al., 2017; White et al., 2017). Liquid-metal-embedded sensors have been utilized in other soft robotics control applications as well (Case et al., 2016; Wirekoh et al., 2019). For this implementation, we use capacitive sensing since it is less sensitive to the change in temperature induced by electrical activation of the SMA actuator. We perform a series of experiments to calibrate the flexural sensor and demonstrate its ability to measure curvature during cyclic actuation. The LM sensor is calibrated for curvature measurement since curvature is the best indicative of a limb actuator state. The actuator always transitions between a fully curved shape with maximum curvature and a relaxed straight shape with minimum curvature. Determining the limb actuator’s curvature contributes to judging if the actuator is fully actuated or fully recovered back to its original curved shape. A simple bang-bang controller is introduced, which utilizes the calibration data and sensor feedback to determine when the actuator has fully relaxed and ready for reactivation. The control strategy is also used to achieve partial actuation within a given range of curvature and evaluate the capability of the actuator to adapt to different environments.

## Background

The nitinol-based SMA actuators exhibit a change shape and stiffness through a temperature-controlled phase change between a malleable twinned Martensite crystal structure and a stiff, shape-restoring Austenite phase (Elahinia, 2015). Because nitinol is conductive, this phase transition can be induced through Joule (Ohmic) heating by applying voltage and passing current through the actuator. In this way, SMA has been used for actuation in a large number of soft robotic systems (Lin et al., 2011; Jin et al., 2016a; Huang et al., 2018, Huang et al., 2019b; Li et al., 2019). However, while promising for these implementations, more widespread application of SMA for robotic actuation is limited by challenges with thermal management and control.

To reduce the accumulation of heat, the SMA wires for the robots are embedded in a thermally conductive elastomer, which passively dissipates heat after each activation cycle. As shown in Figure 1B, the actuator has a large natural curvature in its relaxed state. The embedded SMA wire stiffens and tries to straighten when heated with electrical current, causing the curvature to decrease. It relaxes slowly to its original shape after the power supply is cut off. The frequency of the actuation is primarily determined by how long it takes to achieve fully recovery to its maximal curvature. This deactivation time should be kept to a minimum while simultaneously preventing actuator degradation in a long time period. If activated before the actuator has a chance to cool and fully relax to its original shape, the temperature of the SMA wires will increase so much the wire will eventually lose its shape memory properties. Overheating can be avoided by increasing the time period between activation cycles. However, it is difficult to manually tune an optimal fixed cooling time since these elastomer-based SMA actuators usually require uneven time to relax in different cycles. This required relaxation time not only varies from actuator-to-actuator and cycle-to-cycle, but will also vary with changes in ambient temperature or environmental conditions, e.g., operation in air vs. water.

## Materials and Methods

**FIGURE 2:**
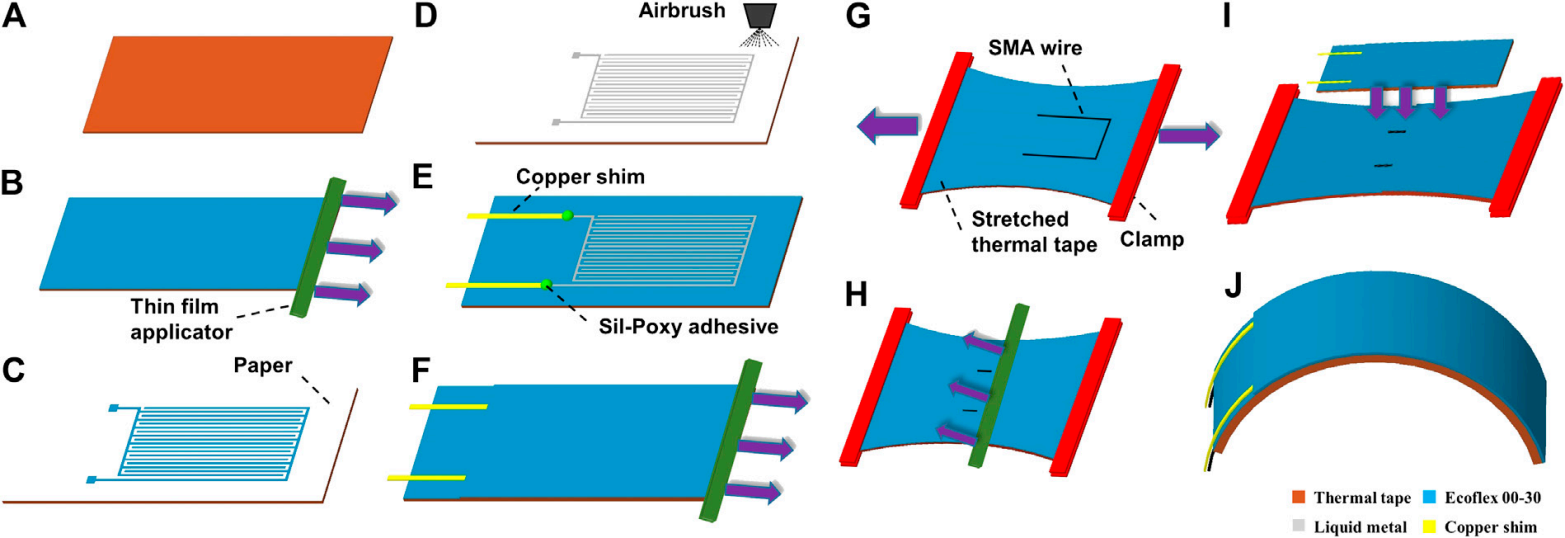
Schematic illustration of the fabrication steps. Left is liquid metal based capacitive strain gague. Right is SMA based actuator. **(A)** Prepare 55×23×0.5 mm thermally conductive elastomer. **(B)** Apply 200 μm thick silicone (Ecoflex) layer on top of elastomer. **(C)** Lay a paper on top of cured Ecoflex; pattern it using a CO2 laser and remove unwanted paper. **(D)** Spray liquid metal on paper mask and peel up the paper mask. **(E)** Put copper shim at each of the two end electrode tabs and secure them using Sil-Poxy adhesive. **(F)** Apply 400 μm thick silicone layer on the sprayed liquid metal. **(G)** Stretch a 80×60×0.5 mm thermally conductive elastomer; apply 100 μm thick layer of Ecoflex on the surface and place a U-shaped SMA wire on the top surface. **(H)** Apply another 100 μm thick layer of Ecoflex on the SMA wire. **(I)** Bond the curvature sensor to the prestretched layer. **(J)** Cut out the actuator after it is fully cured.

Our proposed soft actuator consists of two primary components: the liquid metal capacitive sensor, and the SMA actuator itself. The fabrication process consists of ten steps (Figure 2). The sensor is fabricated first (steps a-f), as described below in *Capacitive Strain Gauge Fabrication* section. The fabrication of the actuator (steps g-j) is described in *Actuator Fabrication* section.

### Capacitive Strain Gauge Fabrication

The EGaIn strain gauge is patterned on a thermally conductive elastomer (H48-2, T-Global) sheet with the dimensions of 55×23×0.5 mm , as shown in Figure 2A. Fabricating the strain gauge on a thermally conductive elastomer makes it easy to be integrated with the SMA actuator. Silicone elastomer (Ecoflex 00-30, Smooth-On) is prepared by mixing prepolymer at a 1:1 ratio by mass in a centrifugal mixer (AR-100, THINKY). Next, a 200  μm thick silicone elastomer layer is applied to thermally conductive elastomer with a thin film applicator (ZUA 200, Zehntner Testing Instruments), as shown in Figure 2B. The thermal tape with uncured silicone coating is cured in an oven at 60 C for 20 min. The thermal tape with cured silicone is then covered with a sheet of plain printer paper that has the same planar dimensions as the thermal tape (Figure 2C).

Next, a CO_2_ laser (30W VLS 3.50; Universal Laser Systems) is used to pattern the paper without damaging the underlying silicone layer, and the excess paper is carefully removed with tweezers (Figure 2C). The laser-patterned paper is utilized as a mask for spray deposition of liquid metal, eutectic gallium-indium, on the silicone layer. The liquid metal is sprayed on the surface with an airbrush (G222-SET, Master) with pressurized argon at about 40 psi. In order to achieve a uniform layer, the spraying process normally needs several seconds while moving the airbrush at a slow speed. The paper mask is then peeled up using tweezers, leaving the patterned LM traces behind (Figure 2D). The result is a comb-shaped LM capacitor with 20 vertical liquid metal combs spaced at 0.4 mm each with a 28 mm length and 0.4 mm width. The combs are oriented vertically to undergo the largest possible change of length since the actuator has the largest deflection along its axial direction when it bends. A larger change in comb length will yield a larger corresponding change in capacitance during bending.

Finally, two pieces of copper shim (50 μm) are placed in contact with the liquid metal tabs as connection electrodes for testing the sensor (Figure 2E). Each piece of copper shim is dipped into liquid metal before being placed on the liquid metal electrode to ensure reliable wetting between the electrodes and copper shims. Next, the pieces of copper shim are securely attached to the thermal tape using Sil-Poxy adhesive, which takes around 30 min to cure. Another 400 μm thick layer of Ecoflex 00-30 is coated on top. After fully curing at 60 C for about 15 min, the capacitive strain gauge is ready to work (Figure 2F). The top sealing layer of silicone is intentionally selected to be thicker to protect the liquid metal trace from leaking and getting damaged when the sensor surface is being touched or in contact with other rigid objects.

### Actuator Fabrication

After cutting out the strain gauge, we are ready to mount it to the outer surface of the actuator. To create the actuator, an 80×60×0.5 mm sheet of thermal tape is clamped at both ends and is stretched around 100% of its length (Figure 2G). The stretched thermal tape is covered with a 100 μm thick layer of Ecoflex 00-30, and then place SMA wire on the top surface. The SMA wire (0.3 mm diameter, resistance of 12.2 Ω/m, Dynalloy) is bent into a U-shape, with leg length of 23 mm and width of 16 mm with pliers. After the SMA wire is placed on the top surface of the stretched thermal tape, it will be covered with another thin layer (100 μm) of Ecoflex 00-30 (Figure 2H).

Next, the prepared capacitive sensor is bonded to the stretched layer by first placing the sensor on the top surface of the stretched layer (Figure 2I). A thin layer of Ecoflex 00-30 is also applied to the bottom surface of the sensor to achieve reliable bonding between the stretched thermal tape and the sensor. The sensor is placed on the stretched layer such that the SMA wire top edge will be a few millimeters away from the top edge of sensor, and SMA wire legs will be located approximately at the same distance from their adjacent sensor side edges.

After the sensor is placed, the stretched layer and the sensor are clamped together with two binder clips. Two soft rubber sheets are used on the top and bottom to avoid direct contact of the binder clips with the sensor and stretched thermal tape. Binder clips apply very high localized pressure to the liquid metal trace and will severely damage the sensor in the case of direct contact. Rubber sheets reduce pressure magnitude and even out pressure distribution. The bonded actuator is placed in a 60 C oven for 20 min in order to become fully cured. Finally, we cut the cured bonded actuator along the outline of the sensor, resulting in a functional actuator with embedded sensor (Figure 2J).

### Experimental Setup

The experimental setup includes the fabricated actuator with the strain gauge, a capacitive sensing chip (MPR121, Adafruit Industries LLC), a microcontroller (Arduino Uno, Arduino LLC), a programmable power supply (KORAD KA3010P, 30V10A), a high-speed camera (GoPro 5), and a laptop (Microsoft Surface Book). A script in Python was used to control the voltage and the actuation time meanwhile collect the capacitance data via serial communication with the power supply and the microcontroller, respectively. The power supply directly provides voltage for activating the actuator. The capacitive chip uses a constant DC charge current scheme for capacitance measurement and communicates with a microcontroller via an I^2^C bus and Interrupt output with a maximum sampling frequency of 100 Hz. In our experiment, the frequency was set as 40 Hz or 60 Hz to make data alignment more convenient as the camera was recording the actuation with a speed of 120 frames per second.

## Results

In order to show that the embedded liquid metal curvature sensor improves overall actuation performance, several experiments were conducted on the actuator. We first performed calibration on our proposed sensor to validate that the capacitance measurement has a good linear relationship with the curvature of actuator. Based on the capacitance measurement, we designed a bang-bang controller to automatically tune the activation time (for the case of partial actuation with limited current) and cooling time (for both partial and full actuation with limited and maximum current, respectively). Then we embedded a miniature thermocouple in the actuator to measure the approximate temperature of SMA wire in the actuator. The temperature verification showed that the closed-loop control strategy was able to help address the overheating problem as well. Finally, we implemented the closed-loop control strategy on the single actuator to achieve locomotion with equal maximum relaxed curvature at each activation cycle.

### Sensor Calibration

The variation in the capacitance of equal coplanar parallel strips that undergo longitudinal stretch was investigated previously (Fassler and Majidi, 2013) and found to be:

$$C=C_{0}\lambda_{x}=C_{0}\left(1+\varepsilon_{x}\right)$$ (1)

In the above equation, C, $C_{0}$, $\lambda_{x}$, and $\varepsilon_{x}$ are the stretched capacitance, prestretched capacitance, ratio of stretched strip length to prestretched strip length, and longitudinal normal strain, respectively. The stretch $\lambda_{x}$ is defined as $L/L_{0}$, where L and $L_{0}$ are the stretched and prestretched lengths of the parallel strips. The strain $\varepsilon_{x}=\lambda_{x}-1$ corresponds to the change in length of the strip when it is being stretched.

Our capacitive comb-shaped strain gauge consists of interdigitated electrodes that get stretched when the actuator bends. In this case, $L_{0}$ is the length of the electrodes when the actuator is straight and L is the stretched length when the actuator bends.

For pure bending, we note that $\varepsilon_{x}=c/\rho =\kappa c$,where c is distance of the outermost surface of the strain gauge from the center of the cross-section of the strain gauge. ρ is the radius of curvature and κ=1/ρ is the actuator’s curvature. Substituting into Eq. 1, yields

$$\begin{array}{c} C=C_{0}\left(1+\kappa c\right) \end{array}$$ (2)

which suggests an affine relationship between the capacitance and curvature of the strain gauge.

**FIGURE 3:**
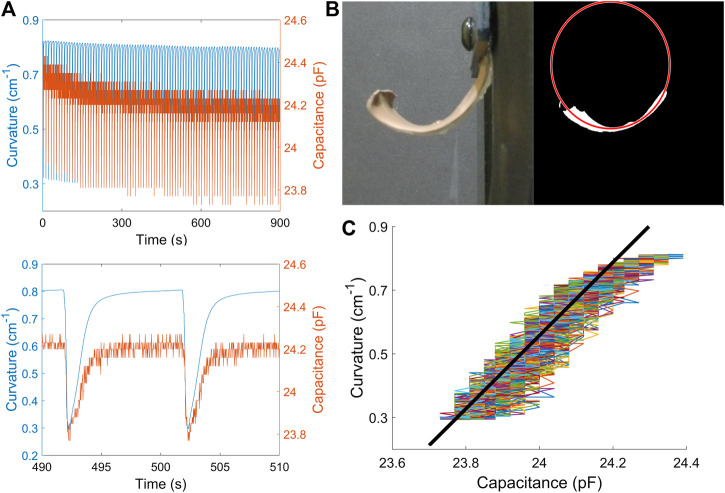
Strain gauge calibration. **(A)** Curvature and capacitance data in time series during one test period **(up)** and two cycles **(down)**. **(B)** The fitted circle using Pratt method in image processing. Curvature is calculated as the inverse of the radius of the fitted circle. **(C)** Linearly relationship between curvature and capacitance during the cooling time. All data were collected under the configuration of voltage as 8 V and activation time as 0.15 s.

In order to verify the relationship between the capacitance and curvature, we measured both capacitance and curvature data (Figure 3A) under different configurations of power voltage and actuation time. The capacitive sensing chip directly read the measurement and transmitted data to laptop through the microcontroller. The curvature was calculated as the inverse of the radius of the fit circle (Figure 3B), processed by MATLAB using the Pratt method (Pratt, 1987). We found that the capacitance decreased nonlinearly with decreasing curvature during the activation period but showed a steady linear response as the actuator returned to its rest curvature during cooling. Several factors, such as edge effects and the fixture at the connection point, might contribute to the noise in the collected capacitance data. The capacitive sensing chip itself additionally had a low frequency (∼2 Hz) white noise. Despite the noise, we were able to align 100 cycles of data into one plot, as shown in Figure 3C. We performed a linear regression on this data with the fitted line (Figure 3C) taking the form of κ= mC+b for curvature κ and capacitance C, with slope m=1.04 and offset b=−24.31. The fit is reasonably linear, with an R^2^ value of 0.914. The linear regression was also performed on the data under different configurations of power supply and activation time (see Supplementary Figure S1) and they all showed a comparatively good linearity, with a standard deviation of slope m equal to 0.08. This calibration result guaranteed the feasibility to implement a closed-loop control strategy for cyclic actuation.

### Controller Design

**FIGURE 4:**
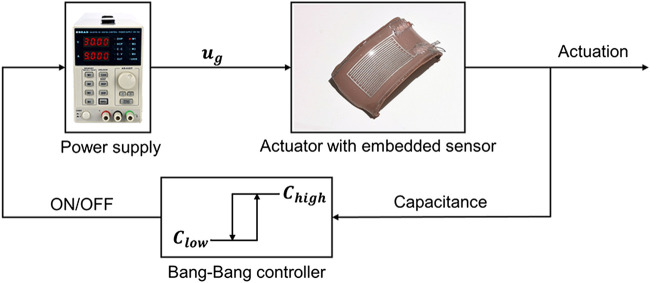
Block diagram of the proposed controller. Both sides are implemented in partial actuation while only the higher bound is used in full actuation.

Based on the calibrated capacitance estimator, we designed a bang-bang controller that used capacitance measurements from the embedded sensor for cyclic actuation. The controller used feedback from the sensor to activate/deactivate the actuator by turning on/off the power supply based on the measured change in actuator curvature (Figure 4). The strategy followed Algorithm 1 with state S and voltage u. The soft limb is either activated (S=A) or cooling down (S=C).

Two variations on the algorithm were used. For the first method, no limit was placed on the current from the power supply, and the controller only determined the starting time of an actuation period. The control system commanded the power supply to apply a given voltage $u_{g}$ to the SMA wire for fixed activation time $t_{A}$. Then the controller waited until the sensed capacitance was greater than a threshold $C_{high}$. For the second method, the current applied to the actuator was limited by the power supply. During activation, the controller followed a similar strategy as during cool-down: voltage was applied to the SMA wire until the sensed capacitance was less than another threshold $C_{low}$. This second approach produced oscillation in an assigned curvature range. Algorithm 1 was implemented in Python and all signal transmission were through serial communication among laptop, microcontroller and power supply.

Algorithm 1 Bang-Bang Cyclical Actuation Controller.

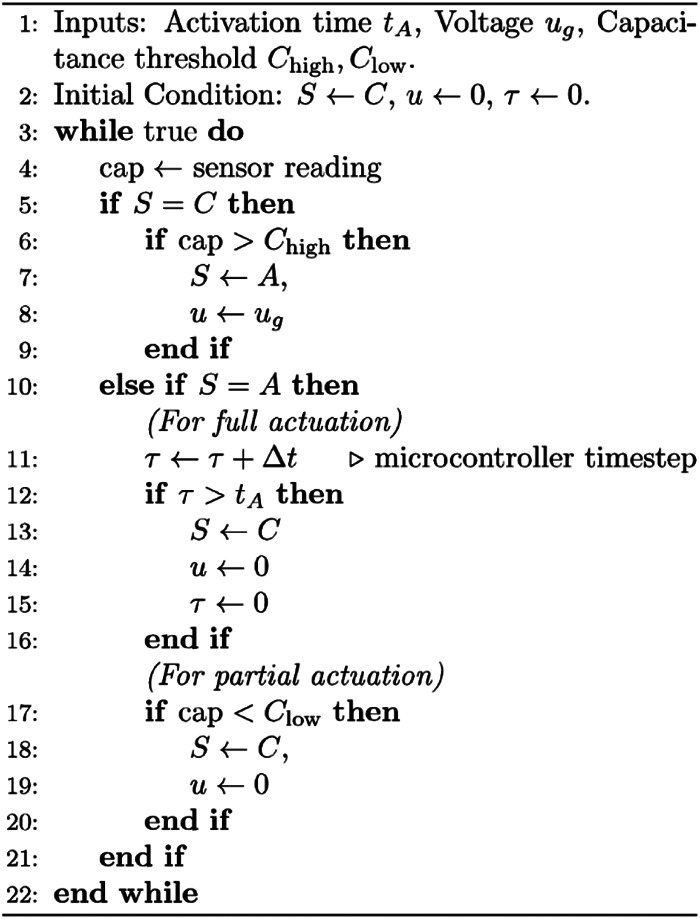

### Full Actuation Characterization

**FIGURE 5:**
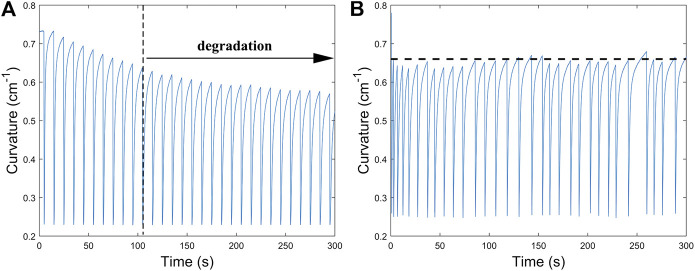
Comparison of curvature range when cycling the actuator, open-loop **(A)** and closed-loop **(B)**. With feedback control that varies the cooling time, the range of curvature does not drift, keeping the actuator relax to a similar position at the start of each cycle. By comparison, the actuator started to degrade at around 100 s with open-loop control strategy.

The control strategy aimed to automatically tune cooling time in each actuation cycle. We compared the actuation performance with open-loop and closed-loop control under the same voltage and activation time (see Supplementary Video S1). From Figure 5A, it is clear that the actuator was gradually not able to recover its natural curved shape with open-loop actuation. The reason is that the length of time required for the SMA actuator to recover its natural shape varied from cycle to cycle and this could not be monitored without sensor feedback. This is due to an increase in time required for the actuator to fully cool at the end of each actuation cycle.

As shown in Figure 5B, we address this with closed-loop control that dynamically modulates the relaxation time. This was accomplished with feedback control using readings from the EGaIn sensor determine when the natural curvature of the actuator was restored. A 30-minute test was performed to demonstrate the long-term stability of the control strategy. The actuator managed to keep its full amplitude during the entire duration of testing. The controller achieved this by gradually adjusting the relaxation time, which increased over the course of cyclic actuation. Full amplitude actuation corresponds to the full range of motion in which the actuator straightens all the way out with near-zero bending curvature followed by complete recovery to the natural curled shape.

**FIGURE 6:**
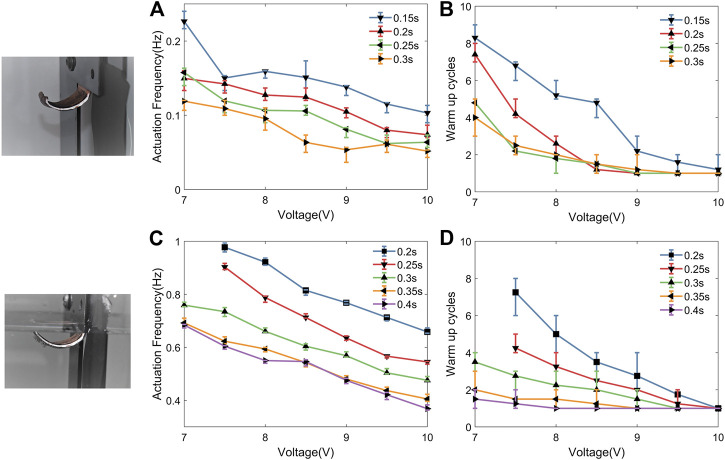
Comparison of actuation frequency and warm-up cycles varying activation time and voltage both in air and water. Normally, high bandwidth with one or two warm-up cycles is optimal for actuator application.

Actuation frequency and the number of warm up cycles were measured both in air (Figure 6A,B) and water (Figure 6C,D) under different configurations of voltage (7–10 V) and actuation time (150–300 ms) as shown in Figure 6, where the data are the averaged results of 5 actuation tests with same configuration. The reason to choose these two essential parameters is to evaluate the actuation performance comprehensively, which we would discuss more details in *Evaluation of Actuation Performance* section. By comparing the results of Figures 6A and 6C, it could be seen that the actuation frequency in water is much larger than in air, even with a longer actuation time. The thermal conductivity is a major difference to explain this phenomenon. The actuator needs much less cooling time in water because water’s thermal conductivity is ∼20 larger than air. Due to this higher thermal conductivity, the actuator needs a longer actuation time or a higher voltage in water to achieve the same full amplitude actuation as in air. From our previous experiments (Huang et al., 2019b), high bandwidth actuation with one or two warm-up cycles is ideal for soft robotics application. Therefore, a 9 V power supply with 150 ms actuation time is identified as well suited for applications in air, while 10 V with 200 ms is best for underwater applications.

### Partial Actuation Characterization

**FIGURE 7:**
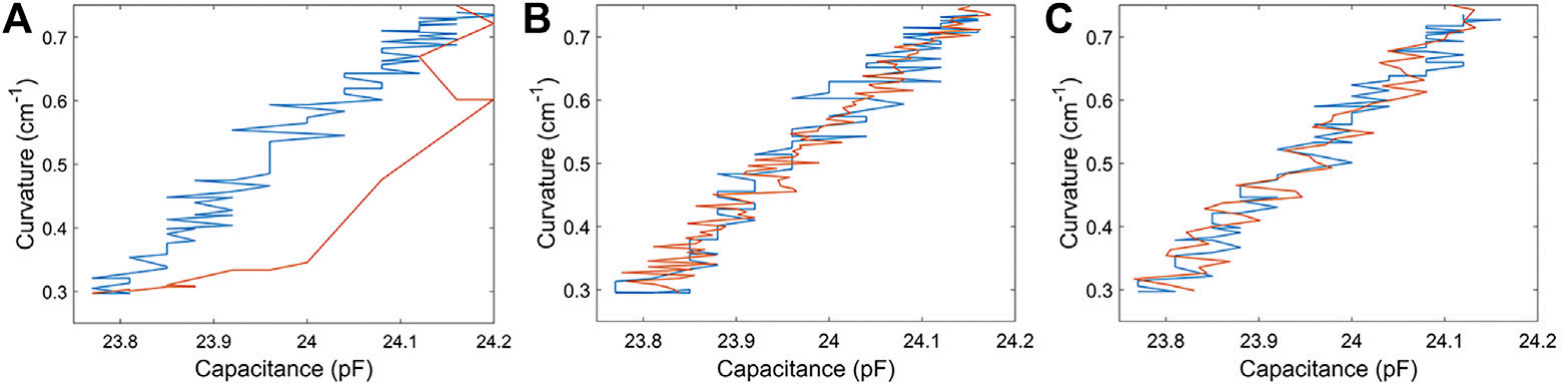
Comparison of calibration result in different current situation. **(A)** Unlimited current actuation. **(B)** and **(C)** are under limited current actuation with constraint of 1.4 A and 2 A respectively. The orange line shows the relationship between measured capacitance and captured curvature in activation period while the blue line in relaxation period.

During the calibration, we found that the capacitance has a large nonlinearity with curvature (Figure 7A) during the activation period. This prevented us from implementing some advanced closed-loop control strategy with sensory feedback during the activation stage of the actuation cycle. The rising temperature of the thermal tape induced by SMA activation did not account for the nonlinearity as the static capacitance measurement was insensitive to the temperature (see Supplementary Figure S2). We therefore postulated that the nonlinearity was primarily due to the relatively fast activation speed of the SMA wire and similarly fast dynamic response of the elastomer. In order to verify our hypothesis, we performed a similar calibration test under limited current, which would make the actuation much slower. As shown in Figures 7B and 7C, reduced current (no more than 2 A) and activation speed allowed the capacitance to show good linearity with curvature in both activation period and relaxation period. Specifically, the R^2^ value of the linear fit is shown to be 0.955 in Figure 7B, and 0.935 in Figure 7C. The result enabled us to implement closed-loop control during the activation stage of an actuation cycle.

As discussed in *Full Actuation Characterization* section, both sides of the bang-bang controller were activated for partial actuation. Instead of having a fixed activation time, the voltage would cut off once the capacitance reached the corresponding lower threshold value. It would continue to power on once the capacitance reached the higher one, which is the same as full actuation. By automatically tuning the activation and cooling times, the controller was able to command the actuator to oscillate in an assigned range of curvatures (see Supplementary Video S2).

**FIGURE 8:**
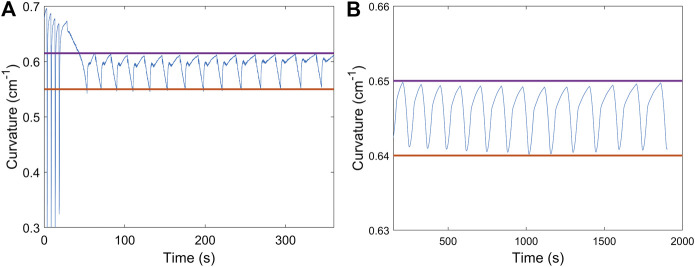
**(A)** Curvature oscillation between 0.55 cm^−1^ and 0.62 cm^−1^ in partial cyclic actuation with limited current (1.4 A). Both sides of the controller were implemented on the actuator to achieve automatically tuning on the activation time and cooling time. **(B)** Curvature oscillation within 0.01 cm^−1^ under the current situation of 0.8 A. The actuator visually maintained at a specific location. In both figures, the two straight line indicates the assigned curvature range in the implementation of the closed-loop control strategy.

As shown in Figure 8A, the actuator began its oscillation with a long period of activation process after several full actuation cycles. The bang-bang controller managed to limit the curvature in both activation and relaxation period respectively. The actuator started or stopped actuation once the capacitance reached the target value. Although not precise for each cycle, the actuator achieved an assigned range of curvature in partial actuation under limited current situation (1.4 A). This range of curvature was manually set so that it could nearly cover the whole span of curvature, though the precision would decrease if the range’s lower bound reached closely to the lowest curvature. Moreover, we reduced the current to 0.8 A (Figure 8B) and managed to make the actuator oscillate within 0.01 cm^−1^ curvature change, which visually seemed to maintain at one specific location.

### Temperature Verification

**FIGURE 9:**
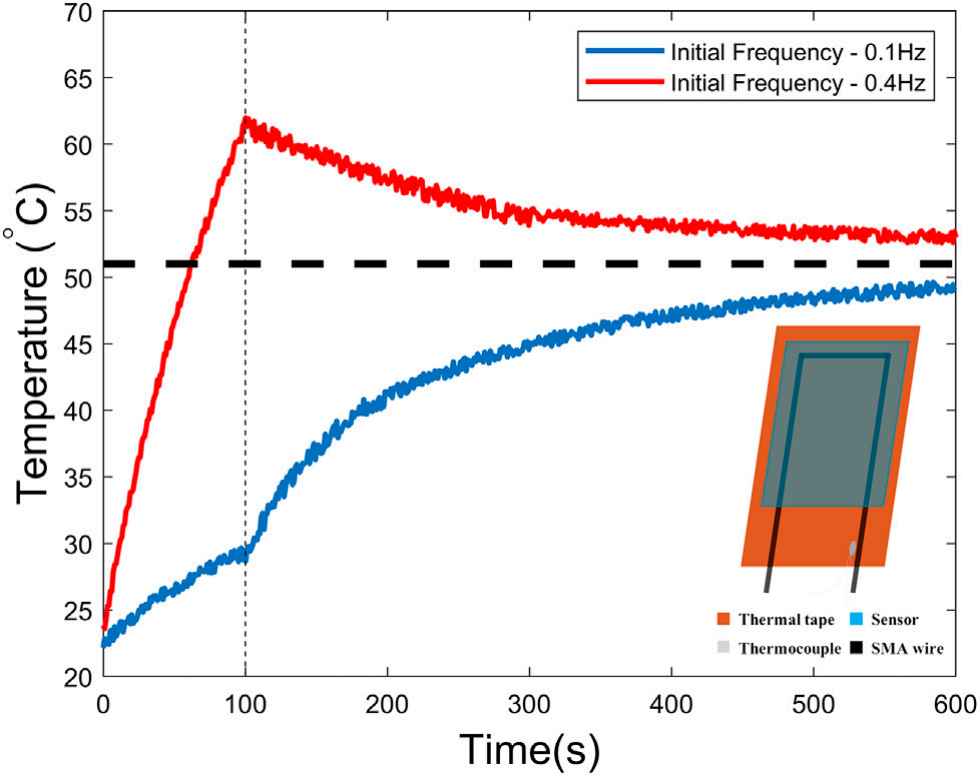
Temperature measurements with different initial open-loop frequency. The closed-loop control strategy is implemented after 100 s. The stable temperature is around 52°C.

Using the method described above, we are able to use closed-loop control to address the issue of overheating. In order to confirm this independently, we perform actuation tests during which we attempt to measure the temperature of SMA wire. However, this temperature is difficult to measure accurately since the wire has a very small diameter (0.15 mm) and the temperature change is rapid during activation period. Therefore, we placed a small thermocouple inside of the actuator, close to one end of the SMA wire as shown in the schematic illustration in Figure 9. Although the thermocouple was not capable of measuring the exact temperature of the embedded nitinol wire, it provided a measurement that scaled monotonically with temperature and could be used to track heating of the nitinol during each actuation cycle.

We measured the temperature during a designed actuation sequence where the actuator was activated under open-loop control with fixed activation time (150 ms) and fixed frequency for the first 100 s and the closed-loop control strategy was implemented on the actuator after that. The strategy was the same as that presented in *Full Actuation Characterization* section, in which the cooling time relied on the feedback of capacitance measurement. As shown in Figure 9, the temperature in the first 100 s increased rapidly with a higher frequency of 0.4 Hz and it implied that the actuator was suffering an overheating problem. After the closed-loop control strategy was used, the temperature started to decrease and finally reached a relatively stable temperature where the actuator had a good performance (curvature approximately ranges from 0.28 cm^−1^ to 0.72 cm^−1^) with a fairly consistent frequency (∼0.24 Hz).

Referring to the blue curve in Figure 9, we did the same experiment again with a lower initial frequency of 0.1 Hz. This time the temperature increased much more slowly. There was no issue with overheating, although we knew that the actuator waited longer than needed in the relaxation period. After implementing the same control strategy, we found out that the temperature increased along with the frequency and eventually reached a stable temperature similar to the previous case. This stable temperature corresponds to the phase transition temperature for SMA actuation. These results imply that the closed-loop control strategy not only helped address the overheating problem to some extent but also tune the optimal frequency automatically.

### Locomotion of Single Actuator

**FIGURE 10:**
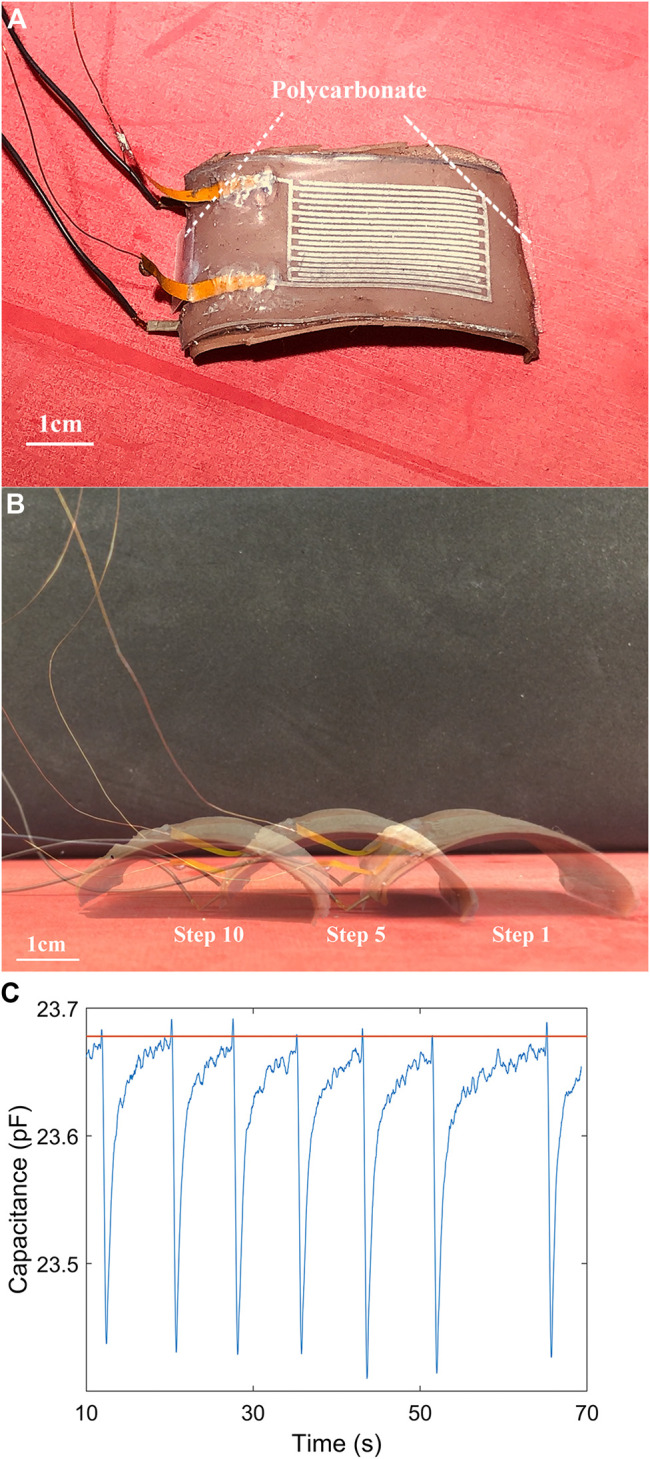
**(A)** A single actuator with two small pieces of polycarbonate attached at both edges. **(B)** Composite of video frames showing the single actuator with auxilary components traveling over 150 s. The curvature is almost the same in three relaxed states. **(C)** The capacitance measurement during a 60-second period of locomotion. The orange straight line is the assigned threshold (23.68 pF) in the control strategy.

The single actuator was able to move on a flat rubber sheet with help of extra auxiliary components (Figure 10A). We attached a small piece of polycarbonate (12×6×0.025 mm) in the front edge and another piece (20×4×0.025 mm) in the rear edge. These two components made the actuator move forward by inducing the imbalance of friction force on two ends. Specifically, when the actuator was relaxing, the rear edge would gradually move forward as the whole actuator slowly recovered back to curled shape (see Supplementary Video S3). Without the front piece of polycarbonate, the front edge would also gradually move backward during the recovery which made the actuator stay at the original location. Therefore, the function of these two pieces of polycarbonate was to make the back edge move smoothly on the flat surface while keep the front edge infeasible to move until an activation occurred.

A real-time closed-loop control strategy was implemented on the locomotion in order to ensure the actuator recovered back to a certain curvature in at the end of each cycle (Figure 10B). The capacitance measurement (Figure 10C) showed that the actuator was not activated until the capacitance reached the assigned value of 23.68 pF which corresponds to a curvature of 0.41 cm^−1^. Theoretically, we were able to select any threshold in the range of performance. However, we found that the measurement would have much noise when the capacitance was larger than 24 pF. When the actuator became more curled, part of the sensor was in contact with the flat rubber sheet, contributing to noise in the capacitive signal. Because of that, we assigned the threshold to be no larger than 24 pF in the experiment, which limited the speed of the single actuator’s locomotion, approximately as 0.01 blps (body length per second).

## Discussion

### Evaluation of Actuation Performance

The control strategy managed to improve the actuation performance for different voltages and activation time but there was not a direct way to choose which one was most suitable for actuation–i.e., achieving full amplitude with the highest actuation bandwidth. Another feature for describing the actuation performance was the number of warm-up cycles, which ranged from 1 to 10 in our tests. Warm-up cycles are defined as the activation cycles required to initiate full amplitude actuation of the SMA actuators. This corresponds to the cumulative heating necessary for the SMA wire to arrive at a temperature close to its phase transition temperature. At the phase transition temperature, the actuator changes from mechanically compliant and curved to stiff and straightened. However, if not enough energy is provided (i.e., the voltage is too low, less than 5 V or the activation time is too short, less than 50 ms), the number of required warm-up cycles would increase. Moreover, if the input energy is too small, the actuator would not achieve full amplitude actuation. In those cases, the warm-up cycles were not counted as it obviously could not be the optimal condition for actuation.

### Potential Improvements and Applications

In general, the sensor fabrication didn’t require extensive materials processing and the patterning step showed good repeatability from sample to sample. However, we found that the stability of the copper electrode tabs had some influence on the sensor performance. Due to its reactivity with metals, gallium-based alloys tend to alloy with other metals like copper and can eventually destroy the metal (Cui et al., 2018; Wang et al., 2019). This concern can be solved by introducing some protective intermediate layer(Morley et al., 2008). In the future, we will consider adding a thin nickel coating on the copper shim.

By enabling the actuator to achieve full amplitude actuation and addressing the overheating challenge, the proposed control strategy has the potential to be utilized for locomotion of SMA-powered soft robots. This includes robots able to crawl, swim and jump (Huang et al., 2019b, Huang et al., 2020) and which are currently operated through open-loop control. Adding embedded sensors could enable tighter control of the robot’s speed and moving direction. Furthermore, such sensors could allow for more complex motion planning for tracking targets of avoiding obstacles.

Moreover, the partial actuation experiments conducted in *Partial Actuation Characterization* section suggest that if we deliver smaller amounts of electrical power, the soft actuator can oscillate within a small window of curvatures. This could make the actuator potentially useful in precise manipulation tasks that require fine motion such as medical robotic devices for operations.

## Conclusion

An SMA actuator with an integrated capacitive liquid metal-based flexural sensor is designed, fabricated, and tested. The capacitance is shown to have a linear relation with the curvature of the soft actuator. The actuator performance is evaluated with open-loop and closed-loop control respectively under the same voltage and actuation time. With the cooling time remaining constant for all activation cycles, the actuator is not able to achieve full amplitude actuation after several cycles since the actuator cannot recover its natural curved shape. In contrast, the actuator is capable of full amplitude actuation with the implementation of feedback control.

To examine the influence of environment on the control scheme, we compared the actuator performance in air and water. The soft actuator frequency in water is larger than in air since the actuator is able to cool down much faster in water. On the other hand, the actuator requires either a longer actuation time or larger activation voltage in water due to the high thermal conductivity of the ambient environment.

For the case of partial actuation, we were able to achieve oscillation in an assigned range of curvature. This was achieved by supplying a smaller amount of electrical current and implementing both sides of the variable structure controller. Partial actuation could have applications in tasks that depend on relatively precise motion control and do not require large force and power density. By embedding a thermocouple into the actuator, we show that the closed-loop control strategy can be used to prevent overheating by allowing the actuator to have adequate time to cool before reactivation.

Lastly, we managed to make the single actuator walk on a flat rubber sheet with attachment of two pieces of polycarbonate. The closed-loop control strategy with real-time capacitance measurement was capable of guaranteeing the actuator to reach the same relaxed curvature at the end of each cycle.

## Data Availability

The raw data supporting the conclusions of this article will be made available by the authors, without undue reservation.
