# Supplementary material for: Shape Memory Alloy (SMA) Actuator With Embedded Liquid Metal Curvature Sensor for Closed-Loop Control
Source: Front Robot AI. 2021 Mar 11;8:599650. doi: 10.3389/frobt.2021.599650 (PMC8059551; doi:10.3389/frobt.2021.599650)
Supplement: Supplementary file 1 [file datasheet1.docx]

Supplementary Material

# Supplementary Figures


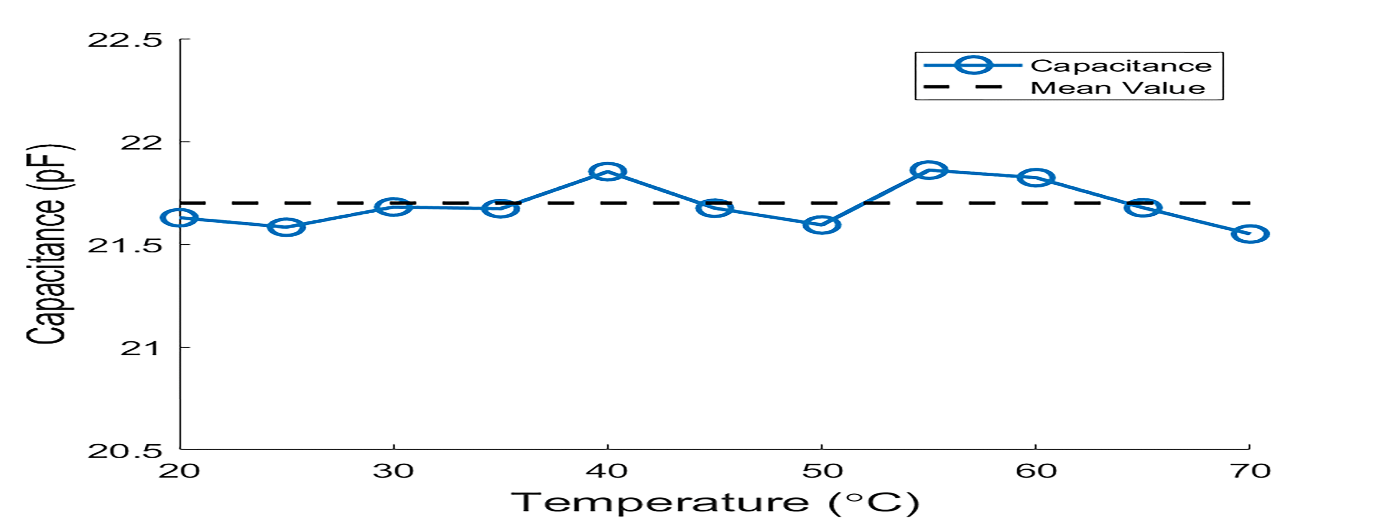


**Supplementary Figure 2.** Capacitance measurement with rising temperature. We placed the sensor on a thermal plate and slowly increased the temperature. With every 5 ℃, we measured 50 capacitance data and calculated the average of them. The measurement showed that the capacitance was insensitive to the temperature.


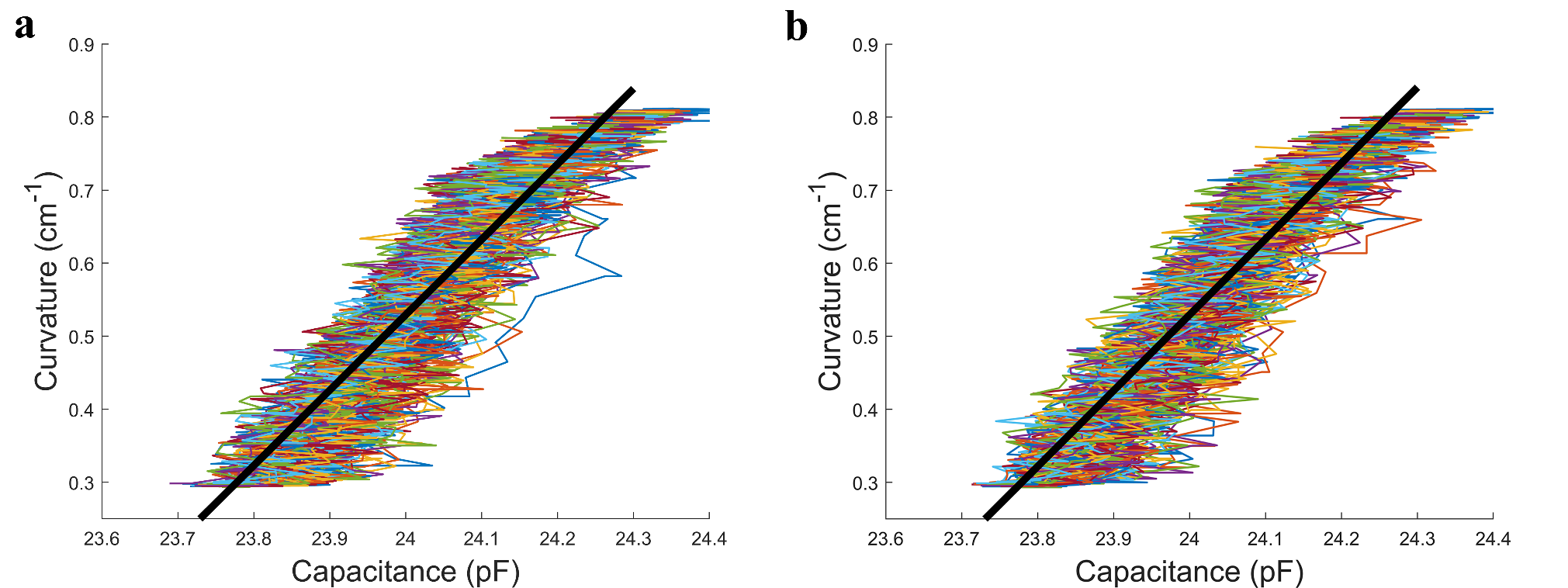


**Supplementary Figure 1.** Capacitance and curvature measurement under different configurations of activation time and voltage. Linear regression was performed on each figure. (a) Voltage as 8 V with activation time as 0.2 s. The fitted line has slope $m=1.03$ and offset $b=-24.23$. (b) Voltage as 9 V with activation time as 0.15 s. The fitted line has slope $m=1.05$ and offset $b=-24.38$.

# Supplementary Videos

**Supplementary Video 1.** Full actuation performance with open-loop and closed-loop control under the same configuration of voltage as 8 V and activation time as 0.15 s.

**Supplementary Video 2.** Partial actuation performance with limited current as 1.4 A. The actuator was able to oscillate between 0.55 cm^-1^ and 0.62 cm^-1^ with “bang-bang” controller.

**Supplementary Video 3.** Locomotion of the single actuator on a flat rubber sheet. The closed-loop control strategy guaranteed the actuator to relax to 0.41 cm^-1^ at each actuation cycle.
